# Effects and central mechanism of electroacupuncture and MRI-navigated rTMS for PSD: study protocol for an fMRI-based single-center, randomized, controlled, open-label trial

**DOI:** 10.3389/fpsyt.2023.1226777

**Published:** 2024-01-04

**Authors:** Hai Lu, Yang Wang, Diwen Shen, Jianguo Ruan, Jiaming Lu, Linlin Wang, Yang Song, Jia Fan, Dongna Li, Lijing Shi, Meng Xia, Tianshu Xu

**Affiliations:** ^1^Department of Traditional Chinese Medicine, Nanjing Drum Tower Hospital, Affiliated Hospital of Medical School, Nanjing University, Nanjing, Jiangsu, China; ^2^Department of Medical Psychology, Nanjing Drum Tower Hospital, Affiliated Hospital of Medical School, Nanjing University, Nanjing, Jiangsu, China; ^3^Department of Radiology, Nanjing Drum Tower Hospital, Affiliated Hospital of Medical School, Nanjing University, Nanjing, Jiangsu, China; ^4^Department of Human Biology, University of Cape Town, Cape Town, South Africa; ^5^Alberta College of Acupuncture & Traditional Chinese Medicine, Calgary, AB, Canada

**Keywords:** electro-acupuncture, MRI-navigated rTMS, post-stroke depression, functional magnetic resonance imaging, randomized controlled trial, study ADL, activity of daily living

## Abstract

**Background:**

Post-stroke depression (PSD) is the most common mental complication after stroke and has a serious impact on functional outcomes and quality of life for stroke patients. Antidepressants are the first-line treatment for PSD; however, many reported side effects remain. Clinical research and practice guidelines have shown that electro-acupuncture (EA) or rTMS have a positive effect on PSD. However, there are few clinical studies on EA and MRI-navigated rTMS for PSD that explore the fMRI-based central mechanism in depression.

**Methods:**

In this randomized, controlled, open-label trial, 64 patients with PSD will be randomly allocated into the experiment group (*n* = 32) or control group (*n* = 32). The experiment group will receive EA and MRI-navigated rTMS and the control group will receive MRI-navigated rTMS treatment, in 12–20 sessions over 4 weeks. In addition, 10 healthy people for fMRI scanning will be recruited as a healthy control group without any intervention. The primary outcome will be the change from baseline in the Hamilton Depression Scale-24 item (HAMD-24) scores at week 4. The primary analysis of the central mechanism will mainly involve cortical morphology, local spontaneous brain activity, and the default mode network (DMN) functional connectivity based on fMRI at 0 and 4 weeks. Secondary outcomes will include the neuro-patho-physiological and quality of life changes in cortical excitability, determined using the motor evoked potential test (MEP), National Institutes of Health Stroke Scale (NIHSS), EuroQol Five Dimensions Questionnaire (EQ-5D) Scale, Modified Barthel Index (MBI) Scale, and Health Scale of Traditional Chinese Medicine (HSTCM). Additional indicators will include the Acceptability Questionnaire and Health Economics Evaluation (cost-effectiveness analysis) to assess the acceptability and economic practicality of the treatment under study. Outcomes will be assessed at baseline and post intervention.

**Discussion:**

EA and MRI-navigated rTMS therapy could become an alternative treatment for PSD, and it is expected that this trial will provide reliable clinical evidence and a potential central mechanism for the future use of EA and MRI-navigated rTMS for PSD.

**Clinical trial registration:**

NCT05516680, ClinicalTrials.gov (registered in August 2022).

## Introduction

Post-stroke depression (PSD), characterized by hopelessness and loss of interest, is a common emotional complication after stroke. Approximately one-third of stroke patients will suffer from depression (1). It has been reported that the incidence of PSD is 4 to 10 times in the common population (2). PSD increases the risk of stroke recurrence/mortality (3) and harms stroke recovery and quality of life (4). In particular, during the COVID-19 pandemic, depression symptoms in patients with stroke increased (5). As we know, PSD is directly related to stroke; however, its underlying mechanisms remain unclear. Moreover, the diagnosis of PSD is mainly assessed using scales, which lack in objective indicators. In addition, because of aphasia and cognitive impairment in stroke patients (6, 7), it is difficult to determine changes in interest and emotion, which makes the early diagnosis and therapy of PSD incredibly challenging. Therefore, it is critical to seek positive and stable predictors for PSD.

Currently, there is still a lack of long-term, safe, and effective therapy for PSD. Based on treatment guidelines for primary depression, anti-depressants are still the clinical first-line therapy for PSD. However, anti-depressants fail to work in approximately 40 to 60% of depressed patients (8), and their onset of effect is usually delayed by 2 to 4 weeks (9). Moreover, several anti-depressants have a risk for cerebrovascular and cardiovascular diseases, leading to low compliance with pharmacological treatment (10). Hence, the need for a safe and effective non-pharmaceutical anti-depression strategy is urgent to prevent depression in patients with stroke.

Acupuncture is a typical, promising, and traditional non-drug therapy, which has been widely used in the treatment of stroke-related diseases in China. Acupuncture, as a national heritage of China, has been recommended for PSD in many international guidelines (11, 12). A network meta-analysis of acupuncture has revealed that, as an adjunctive therapy, it is effective and safety for improving depression in stroke survivors and, combined with repetitive transcranial magnetic stimulation (rTMS), would be a better strategy, with the highest probability (13). The American FDA has approved rTMS as a non-invasive physiotherapy technique, and it is one of the commonly used neuroregulatory techniques for depression in the world. It is reported that publicly funding rTMS for adults with treatment-resistant depression in Ontario over the next 5 years would add $63.2 million in total costs (14). A meta-analysis has shown that patients with PSD may benefit from rTMS (15). Moreover, stroke survivors with depression had positive attitudes toward rTMS (16). rTMS delivers magnetic pulses to stimulate the areas of the brain associated with mood regulation. However, the efficacy of rTMS can be greatly affected by the precise position of the rTMS coil on the scalp (17). In terms of therapeutic target selection, high-frequency rTMS in the left dorso-lateral prefrontal cortex (DLPFC) is often recommended in guidelines to treat PSD (18). However, clinical rTMS typically uses scalp-based landmarks for DLPFC targeting (the ‘5-cm rule’), which cannot achieve accurate positioning because of anatomical differences between individuals. In this study, an alternative method of individual anatomical MRI-guided neuronavigation will be used to accurately locate the DLPFC, so as to realize the focusing and individualization of rTMS targeting. Therefore, acupuncture and rTMS may be a promising choice for PSD.

Few studies have investigated the therapeutic efficacy of acupuncture combined with rTMS, let alone electro-acupuncture (EA) and MRI-navigated rTMS. In addition, the therapeutic central mechanism remains unclear. Existing studies on PSD associated with certain neuro-imaging biomarkers have used resting-state functional MRI (rs-fMRI) based on blood-oxygen-level-dependent (BOLD) signals, and this is a popular and promising method to demonstrate the interactions of brain networks for PSD (19–21). To the best of our knowledge, stroke is often characterized by many changes in the functional connectivity (FC) of neural networks in the brain, which mostly lead to an abnormal emotional circuit, such as the default mode network (DMN) (20). In particular, the DLPFC plays a vital role in the emotional regulation of PSD. Thus, based on rs-fMRI studies, it can help to provide the neuromechanisms and potential targets of the clinical response to EA and MRI-navigated rTMS, building objective criteria to evaluate the therapeutic benefit. In addition, independent component analysis (ICA) will be utilized to assess FC alterations in the DMN, based on the spontaneous low-frequency oscillations (LFOs) of BOLD-fMRI signals representing the brain’s neural networks.

Above all, a randomized, controlled, open-label trial has been designed to investigate the efficacy and central mechanism of EA and MRI-navigated rTMS for PSD by using depression scales and rs-fMRI measurements. Moreover, the improvement of the activities of daily living (ADLs) function of PSD will be also evaluated using related scales.

## Methods

### Design

**Figure 1:**
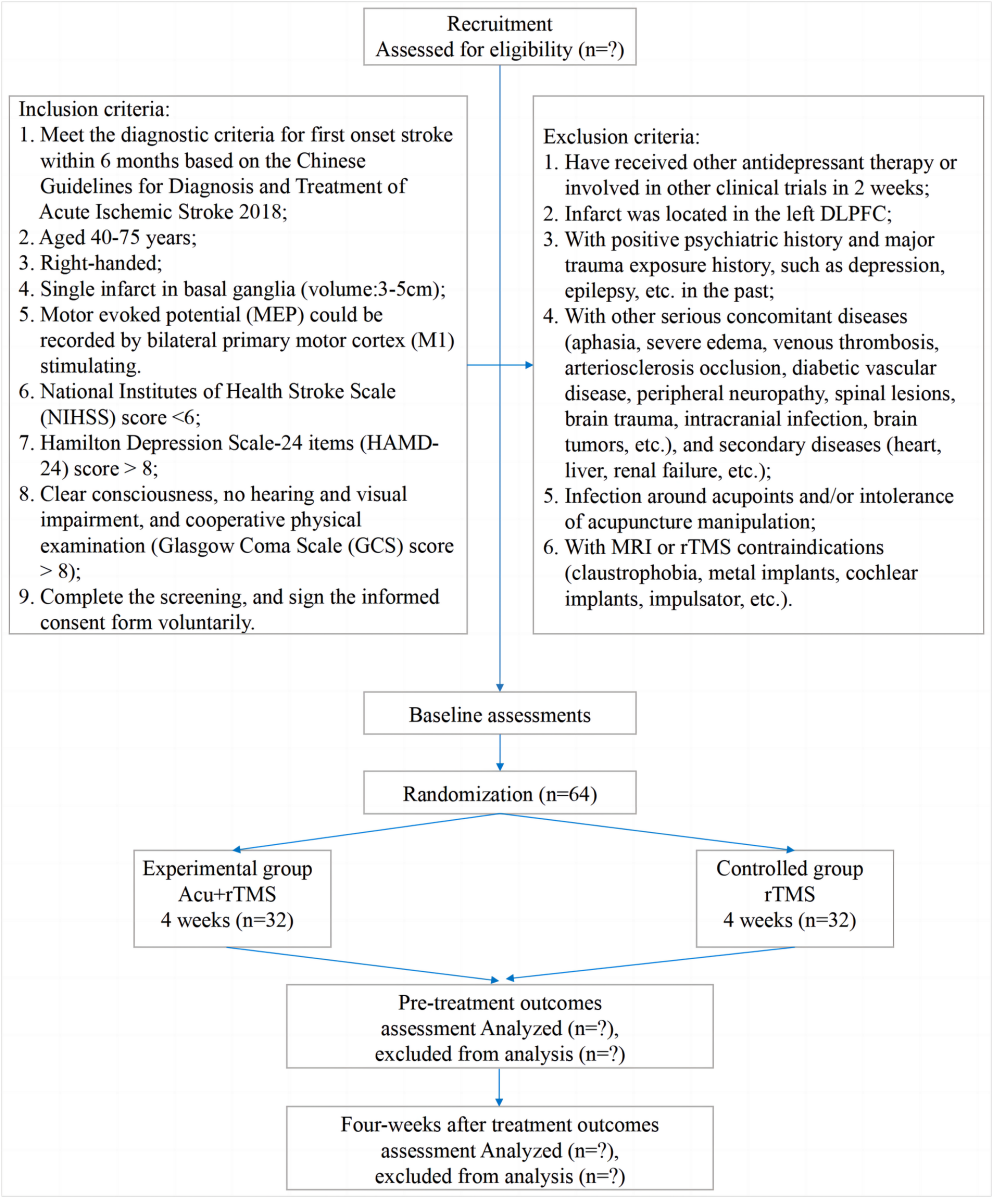
Participant flowchart.

A single-center, randomized, controlled, open-label trial will be performed at Nanjing Drum Tower Hospital, The Affiliated Hospital of Nanjing University Medical School (Nanjing, China). All 64 participants will be randomized into the experimental group (EA and MRI-navigated rTMS) or the control group (MRI-navigated rTMS) at a ratio of 1 to 1. All patients in the two parallel groups will be treated over 12 ~ 20 sessions for 4 weeks (3 ~ 5 sessions per week). The clinical protocol items conform to the SPIRIT 2013 statement (22) and the Revised Standards for Reporting Interventions in Clinical Trials of Acupuncture (STRICTA) (23). The SPIRIT checklist has been presented. The study procedure is listed in Figure 1, and the study schedule is summarized in Table 1.

**Table 1:** Schedule of enrollment, interventions, and assessments.

Study procedure	Study period	
	Enrolment (d)	Treatment period (d)
Time-points	-5th-0d	28th ± 5d
Enrolment		
Demographics	X	
Vital signs	X	
Medical history	X	
Complications	X	
Eligibility screen	X	
MEPs	X	
GCS	X	
NIHSS	X	
HAMD-24	X	
Informed consent	X	
Random allocation	X	
Interventions		
Acu + rTMS	X	
rTMS	X	
Assessments		
rs-fMRI	X	X
HAMD-24	X	X
MEPs	X	X
NIHSS	X	X
MBI	X	X
EQ-5D	X	X
SF-HSTCM	X	X
Acceptability questionnaire		X
Safety evaluations	X	
Patient compliance	X	
Drug combinations	X	
Adverse events	X	
Research summary	X	
Review statements	X	

### Subjects

#### Recruitment strategies

This study will be carried out at the Nanjing Drum Tower Hospital, The Affiliated Hospital of Nanjing University Medical School. All participants will be recruited from the department of TCM, the department of neurology, or the department of psychology. Three doctors (Jianguo Ruan, Lijing Shi, and Diwen Shen) will help to recruit patients and healthy participants from three departments. Recruiting posters and a WeChat platform will be also adopted to collect cases.

### Inclusion criteria

(1) Inclusion criteria for patients include the following:
  1. Meet the diagnostic criteria for first onset stroke within 6 months, based on the Chinese Guidelines for Diagnosis and Treatment of Acute Ischemic Stroke 2018 (24).
  2. Aged 40–75 years.
  3. Right-handed.
  4. Single infarct in basal ganglia (volume: 3–5 cm).
  5. Motor evoked potential (MEP) could be recorded by bilateral primary motor cortex (M1) stimulation.
  6. National Institutes of Health Stroke Scale (NIHSS) score < 6.
  7. Hamilton Depression Scale-24 items (HAMD-24) score > 8.
  8. Clear consciousness, no hearing and visual impairment, and cooperative physical examination (Glasgow Coma Scale (GCS) score > 8).
  9. Complete the screening and voluntarily sign the informed consent form.
(2) Inclusion criteria for healthy subjects include the following:
  1. Healthy subjects, aged 40–75 years.
  2. Right-handed.
  3. HAMD-24 score < 8.
  4. Voluntarily sign the informed consent form.

### Exclusion criteria

(1) Exclusion criteria for patients include the following:
  1. Have received other antidepressant therapy or involved in other clinical trials in the previous 2 weeks.
  2. Infarct located in the left DLPFC.
  3. Positive psychiatric history and major trauma exposure history, such as depression, epilepsy, etc., in the past.
  4. Other serious concomitant diseases (aphasia, severe edema, venous thrombosis, arteriosclerosis occlusion, diabetic vascular disease, peripheral neuropathy, spinal lesions, brain trauma, intracranial infection, brain tumors, etc.) or secondary diseases (heart, liver, renal failure, etc.).
  5. Infection around acupoints and/or intolerance to acupuncture manipulation.
  6. MRI or rTMS contraindications (claustrophobia, metal implants, cochlear implants, impulsator, etc.).
(2) Exclusion criteria for healthy subjects include the following:
  1. Involved in other clinical trials in the previous 2 weeks.
  2. Positive psychiatric history and major trauma exposure history, including depression, epilepsy, etc., in the past.
  3. MRI or rTMS contraindications (claustrophobia, metal implants, cochlear implants, impulsator, etc.).

### Drop-out criteria

1. Low compliance and reluctantly continue to participate in the trial.
2. Unsuitable to continue participation due to serious adverse reactions or complications during the study.
3. Incompliance with standard treatments (less than 50% of The treatment times or receive other unrelated treatments) and/or MRI scanning or incomplete observation data affecting The assessment.

### Intervention

#### Initial therapy

During the 4-week study period, basic treatments for stroke such as antihypertensive agents, anticoagulants and antiplatelet agents, antidiabetic agents, and lipid-lowering drugs, will be allowed to be routinely received. Complication prevention (pressure ulcers and pneumonia) and nutritional support will also be allowed.

### Experimental group

The experimental group will be given EA and MRI-navigated rTMS treatment.

**Figure 2:**
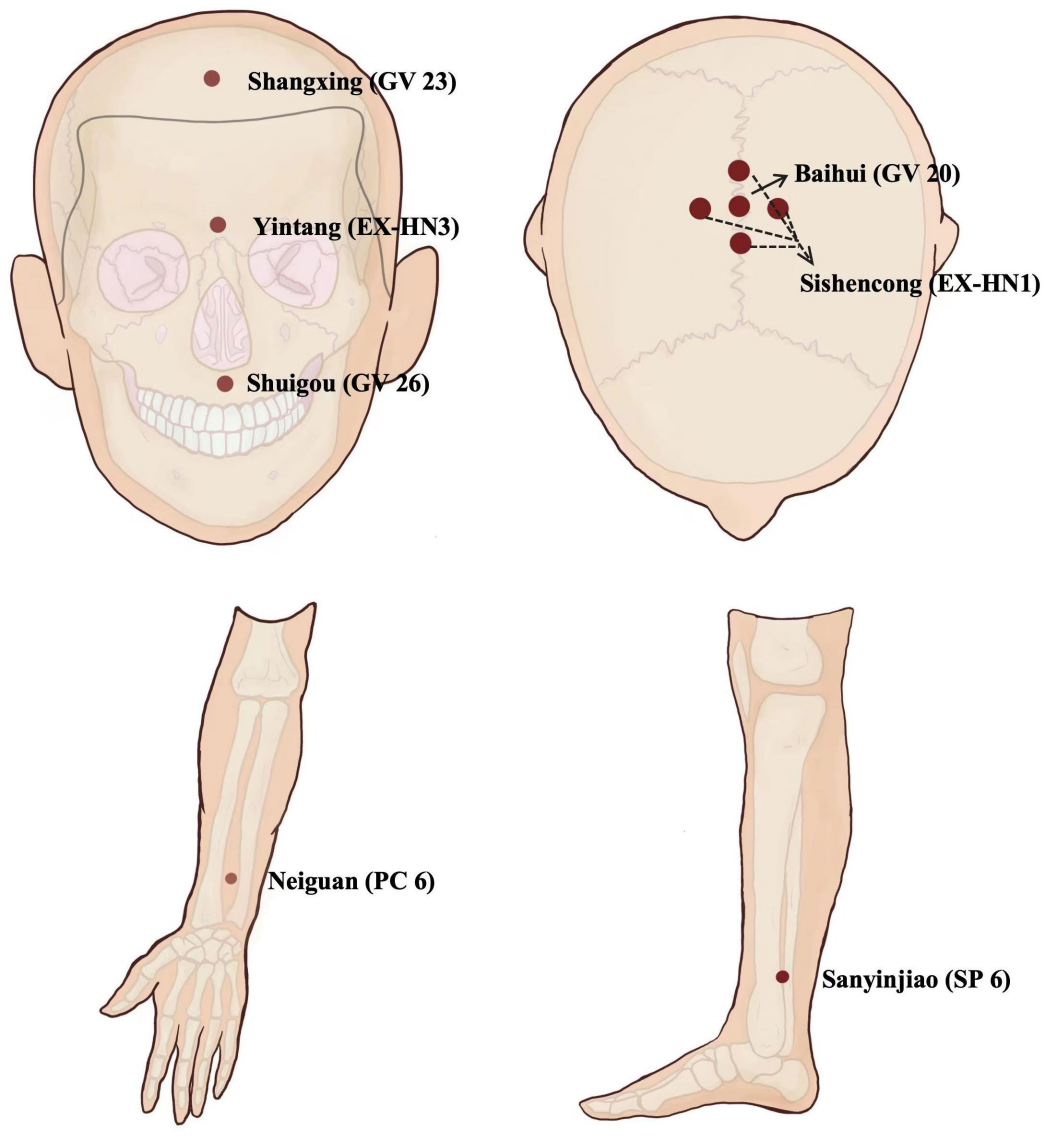
Location of acupoints.

Firstly, EA treatment will be administered at the Neiguan (PC6, bilateral), Shuigou (GV26), Sanyinjiao (SP6, affected side), Yintang (EX-HN3), Shangxing (GV23), Baihui (GV20), and Sishencong (EX-HN1) acupoints. Disposable stainless-steel needles (0.25 mm × 40 mm; Huatuo brand, Suzhou Medical Supplies Factory Co., LTD., Suzhou City, China) will be used. The details of acupoint locations and related acupuncture manipulations have been listed in Figure 2 and Table 2. After skin disinfection, the acupuncturists will twist and thrust the needle handles to achieve the sensations of achiness, heaviness, and numbness (known as de qi) at all abovementioned acupoints. Following needle manipulations, EA instruments (SDZ-V, Hwato brand, Suzhou Medical Supplies Factory Co., Ltd., Suzhou City, China) will be used to attach the needle handles at EX-HN3, GV23, GV20, and EX-HN1, with a dilatational wave of 5 ~ 10 Hz and a current intensity of 1 ~ 3 mA depending on the patient’s tolerance. The needles will be removed after 30 min, except for GV26 without needle retention. Participants will receive 12 ~ 20 sessions over 4 weeks at a frequency of 3 ~ 5 times per week.

**Table 2:** Composition and manipulation of acupuncture.

Acupoints	Location	Manipulation
Neiguan (PC6) (bilateral side)	Pericardium meridian; On the anterior aspect of the forearm, between the tendons of the palmaris longus and the flexor carpi radialis, 2 B-cun (50 mm) proximal to the palmar wrist crease.	Perpendicularly insert approximately 0.5 to 1.0 B-cun (13 mm-25 mm) into the skin, applying combinative manipulation (lifting-thrusting and twirling-rotating) with reducing method bilaterally for 1 min.
Shuigou (GV26)	Governor vessel; On the face, at the junction of the upper 1/3 and lower 2/3 of the philtrum midline.	Obliquely insert upwards to the nasal septum for 0.3–0.5 B-cun (8 mm-13 mm) with heavy bird-pecking method until the patient’s eyeballs have moistened or tears flow.
Sanyinjiao (SP6) (affected side)	Spleen meridian; On the tibial aspect of the leg, posterior to the medial border of the tibia, 3B-cun(75 mm)superior to the prominence of the medial malleolus.	Obliquely insert the point edged the posterior tibia by 45° with skin for 1.0–1.5 B-cun (25 mm-40 mm), and apply tonifying method of lifting-thrusting till the affected side lower limb has jerked 3 times.
Baihui (GV20)	Governor vessel; On the head, 5B-cun(125 mm)superior to the anterior hairline, on the anterior median line.	Obliquely insert toward to back of the head 0.3–0.5 B-cun (25 mm–40 mm) with twirling uniform reinforcing-reducing manipulation for 1 min. Retention for 30 min.
Sishencong (EX-HN1)	Extra point; On the head, 1B-cun(25 mm)around the GV20, and composed of 4 acupoints.	Obliquely insert toward to back of the head 0.3–0.5 B-cun (8 mm–13 mm) with twirling uniform reinforcing-reducing manipulation for 1 min. Retention for 30 min
Shangxing (GV23)	Governor vessel; On the head, 1B-cun(25 mm)superior to the anterior hairline, on the anterior median line.	Subcutaneously insert toward to back of the head 1–1.5 B-cun (25 mm–40 mm) with reinforcing manipulation for 1 min. Twirling manipulation should have amplitude of <90 and frequency of 120–160 turns/ min. Retention for 30 min.
Yintang (EX-HN3)	Extra point; On the forehead, at the midpoint of the line between two brows.	Obliquely insert toward to the nasion 0.2–0.3 B-cun (5 mm-8 mm) with light bird-pecking method until the patient’s eyeballs wetting. Retention for 30 min.

Secondly, MRI-navigated rTMS treatment will include three parts. First, all MRI scans will be taken on a 3 T MRI Scanner with a 32-channel head coil (Philips Achieva 3 T ingenia CX Medical Systems, Netherlands). High-resolution T1-weighted anatomical images will be acquired with TR/TE = 9.8/4.6 ms, flip angle = 8°, FOV = 240 × 240 × 192 mm^3^, matrix = 240 × 240, voxel size = 1 mm isotropic. Second, surface electromyography (EMG) will be adopted to record motor evoked potentials (MEPs) from the abductor pollicis brevis (APB) for determining the resting motor threshold (rMT) of the non-lesioned motor cortex hand representation. A single TMS pulse at 0.2 Hz ± 10% will be delivered to the location to identify the rMT. rMT is defined as the minimum stimulus intensity capable of evoking at least five MEPs of more than 50 μV peak-to-peak amplitude in 10 consecutive trials. The stimulation will be delivered using a Y125-round coil (YRD CCY-II, YIRUIDE GROUP, China). Finally, MRI-navigated, targeted rTMS using a BrainSight system and left DLPFC targeting will be individualized by reconstructing MRI data into a 3-dimensional brain image for neuronavigation. Left DLPFC localization will be the central middle frontal gyrus, between the superior frontal sulcus (BA46) and the middle frontal sulcus (BA9), based on T1-weighted anatomical image data. After navigated localization, focal rTMS will be administered using a Magstim Rapid 2 (Magstim, UK) connected to the identical active Y125-round coil. When treating, rTMS will be applied at 110% RMT to the left DLPFC, with 12 to 20 sessions of 10 Hz (4 s on and 26 s off) rTMS, and a total of 3,000 pulses per session administered 37.5 min per day over the 4-week period.

### Control group

Patients in the control group will only be given MRI-navigated rTMS in the same way as the treatment group.

### Prohibited treatments

Drugs, psychotherapy, transcranial electrical stimulation, music therapy, and other treatments for PSD will be prohibited during the study period. Moreover, acupoints for PSD that are not included in the protocol will also be prohibited.

### Compliance and discontinuation

Based on the above-mentioned withdrawal criteria, if a participant receives more than six EA/rTMS sessions, this will be considered a valid case to generate reliable data. For patients who drop out without enough sessions, their last visit data will be used for result analysis.

### Outcome measures

#### Primary outcomes

The first primary outcome will be the clinical efficacy evaluated by the change of the Hamilton Depression Rating Scale (HAMD) with 24 items (HAMD-24), which meet the scoring criteria as follows: a total score of <8 indicates normal, a total score of ≥8 indicates different levels of depressive symptoms, and a higher score indicates more severe depression. As an other-report and screening questionnaire, individuals with depression will be diagnosed using this scale before enrollment. A HAMD-24 score ≥ 8 will be considered as one of the inclusion criteria in this study. The HAMD-24 score will be assessed at baseline and at 4 weeks.

The second primary outcome will be the change of rs-fMRI in probing possible central mechanisms. Functional neuroimages will be collected using a 3.0 Tesla Philips Ingenia CX MRI system in our hospital. Rs-fMRI data will include three-dimensional T1 weighted structural imaging with a FSE sequence and BOLD imaging with a SE-GRE-EPI sequence for 7.5 min, covering the whole brain. The parameters for 3D-T1* imaging will include a slice thickness of 1 mm, a TR/TE of 9.8 ms/4.6 ms, an FOV of 240× 240 × 192 mm^3^, a flip angle of 8^°^, a matrix of 240 × 240, an in-plane resolution of 1 × 1 × 1 mm^3^, and 192 layers. The parameters for BOLD imaging will include a slice thickness of 4 mm, a TR/TE of 2,000 ms/30 ms, a flip angle of 90^°^, a matrix of 64 × 64, an FOV of 192 × 192 × 140 mm^3^, 35 layers, and 320 time points.

The fMRI data will be preprocessed with the DPABI toolkit.^1^ Preprocessing steps will mainly include slice timing, realignment, regression, normalization, smoothing, detrending, and filtering, similar to a previous study (25).

Regional homogeneity (ReHo), a classical and commonly used analysis in rs-fMRI, provides an indirect measure of the temporal coordination of spontaneous neural activity within a specific region of the brain. Kendall’s coefficient concordance (KCC) can quantify the similarity of a number of time series between a given voxel and the nearest neighbors in a voxel-wise way, using the REST software in Matlab. Specifically, each voxel in the entire brain is compared to its nearest 26 voxels. Subsequently, an individual ReHo image is obtained based on the KCC value, which is computed for each voxel. When normalization is performed, an average ReHo image is achieved by dividing each KCC value by the mean KCC value of the entire brain. The KCC-ReHo value ranges from 0 to 1, and higher values indicate stronger consistency in temporal activity among neighboring voxels and reflects the synchronization of local neuronal activity within the brain.

Amplitude of low frequency fluctuations (ALFF) is another alternative and reliable index of rs-fMRI signals. It has been suggested that it indirectly reflects the regional intensity of spontaneous brain activity in the power spectrum of low-frequency (0.01–0.08 Hz) of the resting-state BOLD signal in the brain, is closely associated with the neural activities, and is physiologically meaningful. ALFF can be divided into distinct frequency bands including slow-5 (0.01–0.027 Hz) and slow-4 (0.027–0.073 Hz). Evidence indicates that fractional ALFF (fALFF) in slow-5 and slow-4 bands could be more sensitive in revealing the features of spontaneous activities in brains compared to ALFF.

The obtained ReHo and fALFF value will be analyzed using MATLAB and SPM12 software. The cluster sizes, active brain regions, and their MNI coordinates will all be noted. In addition, ICA using the GIFT software package^2^ will be used to analyze the data after smoothing preprocessing. This will obtain the spatial distribution pattern of the DMN based on the estimated components, using an informax algorithm during spatial ICA. The results of brain network analysis will be presented in BrainNet Viewer. The abovementioned scanning and analysis will be carried out at baseline and at 4 weeks.

### Secondary outcomes

Motor cortical excitability can be used to predict the response to rTMS treatment applied to the left DLPFC. The cortical excitability of the primary motor cortex (M1) caused by stroke is usually decreased, which can be reflected by the loss of recordable MEPs/decreased MEP amplitudes and increased rMTs. rMT is indicated as a percentage of the maximum stimulator output. M1 cortical excitability will be tested at baseline and at 4 weeks.

The National Institutes of Health Stroke Scale (NIHSS) score is widely used to quantify baseline stroke-related neurological deficits. It is therefore an aid tool to measure stroke severity. The score ranges from 0 to 42 points as a summation of criterionbased integer scores in 11 different domains of neurological function. A greater score suggests a worse outcome (26). In the present study, a NIHSS score < 6 will be considered as one of the inclusion criteria, which will be evaluated at baseline and at 4 weeks.

The modified Barthel index (MBI) is considered to be the best ADLs measurement scale for stroke rehabilitation. The total score for MBI is 0–100, including 10 items. A higher MBI score indicates better ADL function. MBI scores will be measured at baseline and at 4 weeks.

The EuroQol Five Dimensions Questionnaire Scale (EQ-5D) is a widely used instrument developed in Europe that evaluates the generic health status. It shows a simple descriptive profile and a single index value for health care. The minimum and maximum values of the utility index are −0.391 and 1 point, respectively; higher scores mean a better outcome. Furthermore, EQ-5D will also be used in cost-effectiveness analyses for health economic evaluation regarding EA and rTMS treatment in this trial. The EQ-5D scores will be measured at baseline and at 4 weeks.

The short form-health scale of TCM (SF-HSTCM), based on the TCM theory, has been validated as a generic health scale according to subjective feelings in Chinese medicine health care (27). SF-HSTCM is also used to assess the health status of stroke recovery (28). The score of SF-HSTCM ranges from 0 to 130 points, and higher scores mean a worse outcome. The SF-HSTCM scores will be measured at baseline and at 4 weeks.

The acceptability questionnaire is well-designed to assess the acceptability of EA and rTMS treatment, determined through patients’ subjective feelings. It involves three levels: “fully accepted,” “acceptable,” and “not acceptable.” The acceptance rate is calculated via the number of “fully accepted” and “acceptable” subjects. The effective rate is the ratio of effective people to total people. A higher rate means a better acceptability. The acceptability rates will be measured at 4 weeks.

### Sample size

Sample size was calculated based on the literature change of HAMD-24 scores as a primary outcome measure, using the following formula for a comparison between the two groups:


$$n_{2}=\frac{k+1}{k}\frac{{\left(Z_{\alpha /2}+Z_{\beta }\right)}^{2}\sigma^{2}}{\delta^{2}}$$

$$n_{1}=kn_{2}$$

A two-sample *t* test was selected in this research; σ represents the difference between the standard deviations of the HAMD-24 scores at 4 weeks in the experimental group (EA + rTMS) and the control group (rTMS) and δ indicates the difference between the means of the HAMD-24 scores in the two groups. G·Power software (version 3.1.9.7) was used for sample size calculation. According to the results from existing published literature (29), the mean (standard deviation) of the HAMD-24 at baseline before treatment was 27.52 (2.06) for the experimental group and 27.84 (2.55) for the control group. The mean (standard deviation) of the HAMD-24 after 4 weeks of treatment was 11.06 (3.37) for the experimental group and 14.13 (3.15) for the control group. To achieve a power (1 − β) of 0.90 at an alpha level of 5% (2-tailed) and a k value of 1, it was estimated that approximately 25 individuals will be needed in each group. Anticipating a 20% drop-out rate, a total of 64 subjects will be recruited to take part in the study, with 32 patients in each group. Moreover, 10 healthy subjects will be involved in the study, from which normal MRI data will be collected, without randomization and intervention.

### Randomization and allocation concealment

Eligible patients who meet the criteria will be randomly assigned to the experimental or control group at a ratio of 1 to 1, with a randomized block design, namely, A and B. Independent random sequences will be computer-generated and retained by the principal investigator (PI) (Hai Lu), who will not be involved in outcome measurement or data analysis. Therapists will receive each subject’s random number and group assignment through consecutive opaque envelopes. Before participation, the subjects will be informed that they have an equal opportunity to be allocated in either the experimental or control group.

### Blinding

For blinding, because the intervention style will seem dissimilar between EA + rTMS and single rTMS, an open label design will be adopted. However, all subjects will be treated separately to prevent communication in different rooms. Moreover, the MRI scan technicians, outcome assessors, data entry clerks, data administrators, and data statisticians will all be blinded to the grouping with labels A and B.

### Informed consent

The informed consent form has been approved by the Medical Ethics Committee of the Affiliated Drum Tower Hospital, Medical School of Nanjing University (version 1.1). Subjects will be informed of all aspects of the trial, which include the study background and purpose, inclusion and exclusion criteria, available treatments and alternatives, and benefits and harms, as well as rights and duties of the participants. More importantly, informed consent will be obtained from each patient and healthy person before grouping. If patients drop out at any time during the study, the collected data will still be retained for mITT analysis.

### Safety monitoring

Safety indexes will include records of physical examination/vital signs, clinical laboratory tests, and adverse events (AEs) during treatment. If any AEs happen, the investigator will complete the AE form in case report form (CRF), including the onset time, related symptoms/signs, duration, abnormal laboratory examinations, treatment, and prognosis. Then, the investigator will need to evaluate the relationship between the AEs and EA + rTMS based on the form presented in Table 1.

### Data collection and management

During the 4 week clinical study, patients will need to receive EA + rTMS or rTMS alone for 4 consecutive weeks, and two visits will be needed to complete fMRI scans and a series of scales at baseline and at 4 weeks. After screening, the raw data will be comprehensively documented in paper CRF (pCRF) within 2 days and electronic CRF (eCRF) will be completed in the electronic data capture (EDC) system^3^ within 14 days. Nobody, except for the PI (Hai Lu) and clinical research associates (CRA) (Dongna Li) will be eligible to review the database. The raw pCRF documents will be kept in the archives of the Affiliated Drum Tower Hospital, Medical School of Nanjing University for more than 5 years after study completion.

### Statistical analysis

All data analyses of this study will be done by an independent statistician (Jia Fan) from University of Cape Town in South Africa using IBM SPSS Statistics for Windows, version 25.0 (IBMCorp., Armonk, N.Y., USA). The modified intention to treat (mITT) principle will be applied for all outcomes in the full analysis set. If the endpoints are missing, the last-observation-carried-forward (LOCF) method will be used according to mITT analysis. The mean, standard deviation (SD), minimum (min), maximum (max), and median will be reported for the quantitation data. The frequency (*n*) and corresponding percentage (%) will be given for the enumeration data. For intra-group comparisons, Student’s paired t test or Wilcoxon signed rank test will be used, and the chi-squared test will be utilized for qualitative indexes. For inter-group comparisons, independent-sample T test or Mann–Whitney U test will be applied. Pearson correlation coefficient or Spearman correlation coefficient will be used for correlation analysis between scale scores and fMRI data. An alpha level of 5% (2-tailed) will be selected.

### Quality control

With the aim to enhance the feasibility and safety of intervention, one EA therapist (Tianshu Xu) and one rTMS therapist (Yang Wang) with 5 years of experience will be the fixed personnel for the patients, and the MRI scan technician (Jiaming Lu) and outcome evaluator (Yang Song) will probably the same as in the authorization table. Before the study starts, all involved researchers will take several training courses to understand the study protocol, MRI scanning, scales assessment, and the research process. Patient compliance will be a vital factor in the trial. Hence, the following will be conducted throughout the entire study. Firstly, therapists will strictly comply with the informed principle and will assist patients in learning the potential benefits and harms. Secondly, the patients will be required to truthfully complete treatment cards after every treatment. Thirdly, therapists will be able to contact patients using WeChat and phone to provide a kind reminder of the following visit 2 days in advance. In addition, the PI and CRA will strictly carry out three-level quality control management every week, 2 weeks, and 4 weeks throughout the study. The CRA will regularly check the pCRF and eCRF. Traces of modifications will remain. After data entry and verification, the PI will lock the ResMan database for final data analysis, which will be performed by our partner, a professional neuroimaging expert (Jia Fan) from the University of Cape Town, South Africa. The abovementioned measures, to a large extent, will ensure the reliability and repeatability of the research results.

### Trial status

The current study protocol version is 1.0 as of 9 June 2022. After clinical registration, recruitment and randomization began on 22 October 2022. This trial is projected to end on schedule in August 2025.

## Discussion

PSD, a typical neuropsychiatric disorder, is lacking in long-term safe and effective treatments. Therefore, except for antidepressants, it is necessary to find other non-pharmaceutical anti-depression alternatives with safe and effective performance and explore its mechanisms.

In recent years, EA and rTMS have been the most popular alternative therapies for depression and are increasingly used all over the world. A recent randomized controlled trial showed that EA is an efficacious and safe treatment to alleviate the depressive symptoms of PSD and improve neurological function regarding ADLs among stroke patients (30). A new meta-analysis demonstrated that rTMS for PSD also has a positive impact on depression and ADLs compared to antidepressants without any obvious adverse reactions (15). However, few studies were conducted on EA combined with rTMS for the treatment of PSD, and rTMS trials rarely consider individual anatomical MRI-guided neuronavigation.

Depression is the most frequent psychiatric symptom after stroke. Its pathogenesis is associated with lesion of the brain structure and function caused by stroke (31). fMRI studies have revealed that PSD patients tend to be marked by reduced mutual inhibition between functional circuits involving the DMN and nucleus accumbens, as well as volumetric and microstructural changes within these networks (32). It has been found that improvements in depressive symptoms following rTMS are relevant to functional alterations of different brain areas within the DMN of PSD patients (33). The DMN includes the bilateral precuneus, medial prefrontal lobe, anterior cingulate, posterior cingulate gyrus, and inferior parietal lobule, which is mainly responsible for self-consciousness regulation and emotional processing (34). We assume that the psychopathological network for depressive emotion processing deficit in PSD may mainly be associated with the DMN. Existing studies on EA for treating PSD are mostly focused on clinical depressive performance with scales but not brain responses to EA based on fMRI. Hence, a randomized, controlled, open-label trial is designed to explore the efficacy and neuromechanism of EA and MRI-navigated rTMS on PSD. Moreover, we will examine the change of the DMN and how this reflects the emotional function as a special brain network regulator, using ICA measures to provide reliable clinical markers for PSD.

This study does have several limitations. Firstly, it fails to blind patients due to the particularity of EA and rTMS, which may cause performance bias. Secondly, because of limited research grants, the study period only includes the treatment period, without the follow-up period, which makes it impossible to evaluate the long-term efficacy. Moreover, the rs-fMRI results may be influenced by different brain regions of cerebral infarction. The relationship between different infarcts and depression symptoms should be investigated in future studies.

In conclusion, this protocol is strictly reported following the requirements of the SPIRIT statement and the corresponding extension. The results generated from this rigorous design will have high reliability and provide clinical evidence for the underlying benefits and neural correlates of EA and MRI-navigated rTMS for PSD.

## Ethics statement

The studies involving humans were approved by Ethics Committee of the Affiliated Drum Tower Hospital, Medical School of Nanjing University. The studies were conducted in accordance with the local legislation and institutional requirements. The participants provided their written informed consent to participate in this study.

## Author contributions

HL and TX participated in experimental conception and design. HL was responsible for drafting the manuscript. MX was responsible for language and grammar correction. JL was responsible for fMRI scanning. HL, TX, YW, JR, DS, LS, LW, and DL were involved in the study recruitment, intervention, evaluation, and data collection. YS was responsible for data mining. JF was the lead statistician. TX was the supervisor of this study. All authors read this manuscript, approved the final publication of this protocol, and met the four primary ICMJE criteria for authorship.

## Footnotes

^1^ http://rfmri.org/dpabi

^2^ http://icatb.sourceforge.net/

^3^ResMan database, http://www.medresman.org/login.aspx.

## Glossary

ADL: Activity of daily living
AE: Adverse event
ALFF: Amplitude of low frequency fluctuations
BOLD: Blood oxygen level dependent
CRA: Clinical research associates
CRF: Case report form
DLPFC: Dorso-lateral prefrontal cortex
DMN: Default mode network
EQ-5D: EuroQol Five Dimensions Questionnaire
fMRI: Functional magnetic resonance imaging
FC: Functional connectivity
GCS: Glasgow coma scale
HAMD: Hamilton Depression Rating Scale
HSTCM: Health Scale of TCM
ICA: Independent component analysis
KCC: Kendall’s coefficient concordance
MBI: Modified Barthel index
MEP: Motor evoked potential test
mITT: Modified intention to treat
NIHSS: National Institutes of Health Stroke Scale
PI: Principal investigator
PSD: Post-stroke depression
ReHo: Regional homogeneity
rMT: Resting motor threshold
rTMS: Repetitive transcranial magnetic stimulation
